# Antibiotic Resistance Acquisition in the First Week of Life

**DOI:** 10.3389/fmicb.2018.01467

**Published:** 2018-07-04

**Authors:** Olivier Barraud, Marianne Peyre, Elodie Couvé-Deacon, Delphine Chainier, Claire Bahans, Vincent Guigonis, Marie-Cécile Ploy, Antoine Bedu, Fabien Garnier

**Affiliations:** ^1^INSERM, CHU Limoges, UMR 1092, Université de Limoges, Limoges, France; ^2^Service de Pédiatrie, Hôpital Femme Mère Enfant, Limoges, France; ^3^Comité Hme REcherche Clinique, Hôpital Femme Mère Enfant, Limoges, France

**Keywords:** integrons, digestive carriage, antimicrobial resistance, newborn, acquisition

## Abstract

**Objectives:** The fetus is considered sterile but recent studies have suggested that gut colonization could start before birth. Scarce data are available for the acquisition of resistant Gram-negative bacteria (GNB) during the first days of life. Several studies have shown that integrons play a major role in antibiotic resistance acquisition. In this work, we studied the dynamics of human intestinal acquisition of GNB and integrons during the first days of life.

**Methods:** Meconium was collected at birth and a stool sample before hospital discharge (days 2 or 3) on 185 term neonates. GNB were searched by culture on each sample and class 1, 2, and 3 integrons from each GNB or directly from samples. Eight risk factors for integron and GNB acquisition were studied.

**Results:** We isolated 228 GNB, 46 from meconium and the remainder from stools. No link was found between GNB isolation and antibiotic exposure during delivery, but antibiotic exposure during labor significantly selected *bla*_TEM_-positive amoxicillin-resistant *Enterobacteria*. Two-thirds of GNB were antibiotic-susceptible and most of the resistant isolates were acquired after birth. Integrons were detected in 18 of the 228 GNB isolates from 3 meconium and 20 stools. Antibiotic administration during delivery and vaginal carriage of *Streptococcus agalactiae* appeared as risk factors for integron acquisition.

**Conclusion:** Gram-negative bacteria and integrons are mostly acquired after birth during the first days of life even if for some term neonates, meconium was not sterile. Antibiotic administration during delivery is a major risk for integron acquisition and for selection of amoxicillin-resistant *Enterobacteria*.

## Introduction

The fetus is generally considered sterile until the membranes rupture. During vaginal delivery, the infant encounters abundant bacteria in the birth canal and perineal region, and acquires essentially the same microbiota as the mother (Sekirov et al., 2010). This initial microbiota is dominated by aerobic organisms such as *Enterobacteriaceae*, streptococci/enterococci, and moderate levels of staphylococci (Ravi et al., 2015). These microorganisms gradually consume oxygen in the intestine, thereby decreasing local oxidation-reduction potential and thus allowing anaerobic bacteria to become established (Avershina and Rudi, 2013). An adult-like microbiota is established after 3–4 years of life (Sekirov et al., 2010). However, studies suggest that the meconium of term and preterm neonates may not be sterile and that gut colonization may start before birth (Jiménez et al., 2005; Moles et al., 2013).

Antibiotic resistance can occur either through accumulation of mutations or by acquisition of resistance genes. The latter mechanism occurs through horizontal transfer of mobile genetic elements (Martinez, 2014). Together with plasmids and transposons, integrons play a major role in antibiotic resistance acquisition (Ravi et al., 2015). Integrons are able to capture, exchange, and express genes embedded within gene cassettes (Cambray et al., 2010). Different classes of integrons have been defined, based on their associated integrase. Class 1 integrons are the most prevalent in clinical settings. Several epidemiological studies have suggested a link between integrons and multidrug resistance in *Enterobacteriaceae* (Leverstein-van Hall et al., 2003; Nijssen et al., 2005; Daikos et al., 2007; Barraud et al., 2014). Moreover, Ravi et al. (2014, 2015) have suggested that integrons could serve as a reservoir for antibiotic-resistance gene transmission within the intestinal microbiota.

Only a few studies have analyzed the gut colonization of resistant bacteria in the first week of life.

The aim of this work was to study the dynamics of human intestinal acquisition of Gram-negative bacteria (GNB) and integrons during the first day of life, based on analysis of meconium and of stools collected just before hospital discharge.

## Materials and Methods

This prospective observational study took place in the maternity unit of Limoges teaching hospital (France) between October and November 2010. With their parents’ written informed consent, we enrolled all neonates born at 36 weeks of pregnancy or more. The Medical Research Ethics Committee of the Limoges hospital approved the study (ref. 51-2010-09). Clinical data were collected, namely *Streptococcus agalactiae* carriage, mode of delivery, premature rupture of the membranes, colored gastric fluid, time since membrane rupture, antibiotic exposure during delivery, feeding mode, and gestational age.

Meconium was collected at birth and a stool sample just before hospital discharge (days 2 or 3). Samples were inoculated onto Drigalski agar plates (BioMérieux, Marcy l’Etoile, France) and incubated for 48 h at 35–37°C. Bacterial identification and susceptibility testing were done with the Vitek 2^®^ system, using the ID-GN^®^ and AST N-233^®^ cards, respectively, (BioMérieux, Marcy l’Etoile, France) as recommended by the manufacturer.

Total DNA from stool samples was extracted with the QIAamp^®^ DNA Stool Mini kit (Qiagen^®^) and used for qPCR detection of class 1, 2, and 3 integrons, as previously described (Barraud et al., 2010). For integron and beta-lactamase genes detection from GNB, a thick suspension was prepared in 1 ml of sterilized water and incubated at 100°C for 20 min. After centrifugation at 10 000 rpm for 10 min at 4°C, the supernatant was used for PCR detection. The gene cassette content of class 1 and class 2 integrons was determined, as previously described (Gassama Sow et al., 2010). The *bla*_TEM_ and *bla*_CTX-M_ genes were detected and sequenced with primers TEM-A /TEM-B and CTX-M A/CTX-M B as previously described (Brasme et al., 2007). Fisher’s exact test for nominal and categorical variables, or the chi-squared test were used as appropriate. Significance was set at *p* < 0.05. All statistical analyses used Epi Info version 3.3.2.

## Results and Discussion

We included 185 newborns delivered by 184 mothers. One hundred and eighty-five meconium samples and 192 stools were collected (two stools were collected from seven newborns).

Delivery was by cesarean section in 43 cases. During labor, 47 women received antibiotics (44 amoxicillin, 2 spiramycin, and 1 cefixime), for *S. agalactiae* vaginal carriage in 22 cases, for a time since membrane rupture of more than 6 h in 19 cases, for fever in 1 case, and for unknown reasons in 5 cases. The gastric fluid was colored in 18 cases (9.8%). Neonatal feeding was maternal in 121 cases, artificial in 48 cases, and mixed in 16 cases. The average length of hospital stay was about 4 days.

We isolated a total of 228 GNB (**Supplementary Table S1**), 46 from meconium of 42 newborns (22.7%), and the remainder from stools (149 newborns, 80.5%). GNB were only isolated from meconium in case of vaginal delivery, possibly because of delayed fecal colonization in infants born by cesarean section (Long and Swenson, 1977). We found no link between GNB acquisition and antibiotic exposure during delivery (*p* = 0.05, **Table 1**).

**Table 1:** Risk factors for integron and Gram-negative bacteria (GNB) acquisition.

	Total (*n*)	Integron acquisition	*p*	GNB acquisition	*p*
*S. agalactiae* carriage: Positive	22	7	0.043^∗∗^	21	0.050^∗∗^
Negative	158	13		122	
Vaginal delivery	142	15	0.785^∗∗^	124	<0.001^★^
Cesarean section	43	5		20	
Premature rupture of membranes: <6 h	128	13	0.667^★^	97	0.312^★^
≥6 h	57	7		47	
Gastric fluid; colored and/or meconial	18	3	0.419^∗∗^	11	0.080^∗∗^
Clear	167	17		133	
Membrane rupture: <6 h	124	10	0.079^★^	99	0.320^★^
≥6 h	60	10		44	
Antibiotics during delivery: No	138	11	0.033^★^	107	0.865^★^
Yes	47	9		37	
Feeding: maternal	121	14	1^∗∗^	95	0.850^∗^
Artificial	48	5		36	
artificial + maternal	16	1		13	
Gestational age: <39 weeks	57	7	0.667^★^	42	0.364^★^
≥39 weeks	128	13		102	

^∗★^*Test used was the chi-squared test, ^∗∗^Test used was the Fisher’s exact test, n, number of patients.*

The main GNB isolates were *Escherichia coli* (76.3%), *Klebsiella pneumoniae* (5%), *K. oxytoca* (4%), and *Enterobacter cloacae* (4%). GNB (mainly *E. coli*) were recovered from 58% of newborns. Previous studies have shown that the microbiota evolves rapidly after birth and that *E. coli* is the first colonizer of the infant gut (Jiménez et al., 2008). We found that *Klebsiella* was the second member of the *Enterobacteriaceae* to colonize the gut, whereas Jiménez et al. (2008) found it was *Enterobacter*. This difference could result from different ecologies between Spain and France.

Two-thirds of GNB isolates were antibiotic-susceptible (**Supplementary Table S1**). Among the one-third of isolates (*n* = 75) resistant to at least one antibiotic family, most of them acquired a *bla*_TEM-1_ penicillinase and only five isolates (2.2%), recovered from four newborns, were resistant to third-generation cephalosporins: two expressed an ESBL (*bla*_CTX-M-15_) and three a hyperproduction of cephalosporinase. This low rate of resistance matches the ecology of our maternity unit (data not shown). Only one mother of these four newborns received antibiotics during labor. Importantly, most of the isolates with acquired resistances (79%) were acquired after birth, probably from the mother or from the hospital environment. In the absence of maternal sampling, we are unable to answer this question. Ravi et al. (2015) also failed to identify the source (hospital environment or mother) of bacterial transmission at an early age, despite using whole-genome sequencing. Interestingly, antibiotic exposure during delivery significantly selected amoxicillin-resistant *Enterobacteriaceae* strains (39/56 vs. 75/168, *p* = 0.001).

We searched for integrons directly in the meconium and stool samples and in the GNB isolates. Integrons were detected in 3 (1.6%) of the 185 meconium samples and 20 (10.4%) of the 192 stools, from 20 newborns (10.8%). All integrons detected in meconium were also detected in the corresponding stool. At least one integron was found in 18 (7.9%) of the 228 GNB isolates, with *E. coli* representing 78% of integron-positive isolates (**Supplementary Table S1**). Class 1 integrons were the most prevalent (*n* = 16), followed by class 2 integrons (*n* = 2). No class 3 integrons were detected. Most gene cassettes encoded resistance to trimethoprim (*dfrA*) and streptomycin/spectinomycin (*aadA*) (**Supplementary Table S1**). These results are in keeping with previous studies, in which class 1 integrons predominated (Cambray et al., 2010; Vinué et al., 2010). The results for integron detection directly in meconium and stool samples and in GNB isolates agreed in 60% of cases. By contrast, integrons were more detected in stools than in bacterial isolates, possibly because non-cultivable bacteria can also contain integrons or because some bacteria may not have been collected during the culture process as already described (Chainier et al., 2017). Moreover, it has been shown that Gram-positive bacteria, especially Corynebacteria, may contain integrons (Nešvera et al., 1998; Nandi et al., 2004; Barraud et al., 2011). However, our method using Drigalski agar plates only allowed isolating GNB. Using the cultivation-independent method, these Gram-positive bacteria containing integrons may have been detected. Lastly, bacteria from patients under antibiotics may have been unable to grow. It is noteworthy that 14 (77.8%) of the 18 integron-positive isolates were markedly resistant to trimethoprim-sulfamethoxazole (*p* < 0.001), due to the presence within the integron of the frequent *dfr* gene cassettes, which encode resistance to trimethoprim and to the *sul1* gene which encodes resistance to sulfonamides located in most class 1 integrons.

A recent study (Ravi et al., 2015) showed a prevalence of 15% of integrons at 3–10 days and 4 months of age, with high-level persistence until 2 years of age. Our findings are in keeping with these results. Integron carriage in newborns seems lower than among adult subjects for which values are higher (Chainier et al., 2017). This could suggest that integron acquisition occurs throughout life. Our detection of GNB and integrons in meconium is in line with previous studies suggesting that gut colonization can start before birth (Jiménez et al., 2008; Moles et al., 2013). Several previous studies have suggested that bacteria may spread transiently through the bloodstream from the maternal digestive tract to extra-intestinal sites in the newborn (Kornman and Loesche, 1980; Jiménez et al., 2005; DiGiulio et al., 2008). Unfortunately, our study did not include maternal sampling. Further studies that include maternal sampling (stools and/or vaginal swabs) will be necessary to determine whether bacteria acquired by neonates during the hospital stay come from the mother or from the environment.

Antibiotic administration during delivery was the main risk factor for integron acquisition (*p* = 0.033, **Table 1**). Surprisingly, a link between integron acquisition and vaginal carriage of *S. agalactiae* was also found (*p* = 0.043, **Table 1**). However, these two risk factors are linked, as antibiotics are recommended to prevent *S. agalactiae* infection of the newborn. Although intrapartum antibiotic prophylaxis did not increase the GNB isolation rate, amoxicillin exposure significantly selected amoxicillin-resistant GNB, as already reported (Edwards et al., 2002).

Fecal metagenome studies could improve the detection of integrons, GNB, and multidrug-resistant bacteria (Andersen et al., 2016). Such type of analysis could help us in deciphering the microbiome and resistome of newborns.

Our study showed that integrons are acquired early in the first week of life and that antibiotic administration during delivery is a major risk for integrons acquisition.

## Author Contributions

OB and EC-D performed analyses and proofreading of the manuscript. MP included the patients, recovered the data and the experiments. DC and CB performed analyses and recovered the data. VG and AB included the patients and proofread the manuscript. M-CP wrote the manuscript and proofread the manuscript. FG performed analyses, wrote the manuscript, and proofread the manuscript.

## Conflict of Interest Statement

The authors declare that the research was conducted in the absence of any commercial or financial relationships that could be construed as a potential conflict of interest.
